# Horizontal mapping of time-related words in first and second language

**DOI:** 10.1038/s41598-024-60062-1

**Published:** 2024-04-27

**Authors:** Anastasia Malyshevskaya, Martin H. Fischer, Yury Shtyrov, Andriy Myachykov

**Affiliations:** 1 Potsdam Embodied Cognition Group, Cognitive Sciences, University of Potsdam, Karl-Liebknecht-Str. 24/25, 14476 Potsdam-Golm, Germany; 2 Centre for Cognition and Decision Making, Institute for Cognitive Neuroscience, HSE University, Krivokolenniy Pereulok 3, Entrance 2, 101000 Moscow, Russian Federation; 3 Center of Functionally Integrative Neuroscience (CFIN), Institute for Clinical Medicine, Aarhus University, Universitetsbyen 3, Bldg. 1719, 8000 Aarhus, Denmark; 4 Department of Psychology, Northumbria University, Northumberland Building, Newcastle Upon Tyne, NE1 8ST UK

**Keywords:** Human behaviour, Psychology and behaviour

## Abstract

The existence of a consistent horizontal spatial-conceptual mapping for words denoting time is a well-established phenomenon. For example, words related to the past or future (e.g., yesterday/tomorrow) facilitate respective leftward/rightward attentional shifts and responses, suggesting the visual-spatial grounding of temporal semantics, at least in the native language (L1). To examine whether similar horizontal bias also accompanies access to time-related words in a second language (L2), we tested 53 Russian-English (Experiment 1) and 48 German-English (Experiment 2) bilinguals, who classified randomly presented L1 and L2 time-related words as past- or future-related using left or right response keys. The predicted spatial congruency effect was registered in all tested languages and, furthermore, was positively associated with higher L2 proficiency in Experiment 2. Our findings (1) support the notion of horizontal spatial-conceptual mapping in diverse L1s, (2) demonstrate the existence of a similar spatial bias when processing temporal words in L2, and (3) show that the strength of time-space association in L2 may depend on individual L2 proficiency.

## Horizontal mapping of time-related words in first and second language

Cognitive research provides numerous examples of regular and consistent spatial biases accompanying processing of both concrete and abstract concepts [see^1,2,3^ for meta-analyses and reviews]. For example, processing words with implicit concrete spatial referents, e.g., *bird* and *root*, induces corresponding systematic upward and downward attentional shifts^4–7^. Similar spatial biases accompany the processing of abstract concepts, e.g., words with emotional^8^, numerical^2^, and temporal semantics^9^. For example, passive listening to number names induces leftward ocular drift for smaller numbers and rightward ocular drift for larger numbers^10,11^. These and other examples indicate that the relationship between visuospatial and conceptual processes is regular and robust, providing general support to the embodied/grounded theories of cognition that emphasize the intrinsic link between perceptual experiences and high-level cognitive functions^12^. Yet, the ubiquity of this association across conceptual domains and different languages, as well as the functional neurocognitive systems supporting it, remains understudied. The present study focuses on the horizontal attentional displacement known to accompany the processing of *time-related concepts*—highly abstract and very common across languages^13^.

Previous findings indicate that the processing of temporal words in one’s *native* language (L1) is regularly accompanied by visuospatial biases^14–16^. These biases manifest as a complex three-dimensional space whereby individual time concepts related to the future or the past can be located along sagittal, vertical, and horizontal axes^17,18^. Among these three axes, or *Mental Time Lines (MTLs)*^9,14^, the *sagittal* MTL is most commonly observed in space-time metaphors such as *past is behind* or *future is in front*^15,19^. Indeed, speakers of different languages associate past events with backward and future events with forward space [but see^20–24^ for culture-specific variations]. The regular and largely universal orientation of the sagittal MTL is likely to result from the regular sensorimotor experience of moving forward in space^14^. Considerable research supports this view, showing facilitation of processing past/future related information while performing backward/forward movements in space, respectively^18^, e.g.,^25^.

In contrast, the *vertically* oriented MTL is likely to reflect cultural and context-specific biases expressed in, e.g., spatio-temporal metaphors^26,27^,but see^28^. For example, speakers of languages that emphasize vertical dimension for time metaphors (e.g., Mandarin: [shàng zhōu] (“above week”) meaning *last week* and [xià xià zhōu] (“below week”) meaning *the week after next week*) were shown to associate past-related information with upward and future-related information with downward space but see^18,27,29–31^. This pattern might not necessarily reflect a broader cultural influence but could be a result of habitual linguistic practices within specific settings or contexts. On the other hand, representatives of European languages without vertical spatio-temporal metaphors demonstrate a reverse pattern, with their vertical MTL oriented from the bottom to the top^32,33^. This variation suggests that the orientation of vertical MTL can be significantly influenced by conventional practices and preferences in language use, which are shaped by the linguistic environment and the specific contexts. That is, while cultural factors do play a role, the specific sociolinguistic context, including the habitual use of language and exposure to specific lexical choices, appears to exert a more direct influence on the vertical MTL.

More relevant for the research reported here is the *horizontal* mapping of temporal events and time-related words^9,34,35^ for reviews. Indeed, a left-to-right mapping of temporal concepts representing progression from past to future was shown to emerge early in linguistic development^36–40^. In adults, processing of past and future-related temporal expressions was shown to induce horizontal spatial biases in pointing gestures^41^ [but see^23,42^ for some exceptions], eye movements^43^, and manual responses e.g.,^44,45^. The notion of a horizontal MTL is supported by the existence of the so-called *Spatial–Temporal Association of Response Codes (STEARC)* effect: Left-oriented responses are typically faster when processing past-related words while right-oriented responses are faster when processing future-related words e.g.,^44,45^.

As with the vertical axis, the orientation of one’s horizontal MTL may be modified by cultural effects, e.g., reading direction habits differing across languages^14,28,46,47^. Indeed, the “classical” left-to-right orientation of the MTL has been confirmed for left-to-right reading and writing systems^48,49^ while its reversed direction has been documented for cultures with right-to-left reading and writing habits^47,49,50^. Thus, MTL research provides compelling evidence for a regular but flexible mapping between temporal concepts and the horizontal space that depends on an individual’s linguistic and cultural habits.

While the evidence regarding the activation of a horizontal MTL during processing of temporal words in an individual’s L1 is quite consistent, it remains largely unclear to what extent similar spatial biases accompany the use of this and other types of abstract concepts in one’s second language (L2), since previous findings show inconsistent results [see^51,52^ for reviews]. Note that L1 acquisition represents a long-term process involving numerous interactions with other people and the physical world, thus naturally producing sensorimotor grounding of word meanings. Instead, the acquisition of L2 (at least for late bilinguals) relies mostly on didactic instruction, often relying on translation. Moreover, L2 is often used in more limited contexts than L1, e.g., only in academic or professional environments. As a result, late acquired L2 might be viewed as less (or even not at all) grounded in regular sensorimotor experiences when compared to L1^53^, thus leading to weaker MTL activation.

Nevertheless, some studies using Mandarin-English (ME) bilinguals have demonstrated a complex interplay between visuo-spatial mapping of temporal information in L1 and L2 in a three-dimensional space^18,26,30,54–56^. For example, several studies showed simultaneous activation of the vertical (commonly observed in Mandarin speakers) and the horizontal (commonly observed in English speakers) axes in ME bilinguals during processing of temporal information e.g.,^18^. Furthermore, ME bilinguals were shown to adopt either time- or ego-moving perspectives of time representation (e.g., *the deadline is approaching* vs. *we are approaching the deadline*), depending on processing L1 Mandarin or L2 English spatio-temporal metaphors^55^. At the same time, research directly comparing differences between L1 and L2 with regard to the horizontal mapping of temporal words is sparse. Specifically, only a few studies have compared differences in the processing of temporal information in L1 vs. L2 in languages beyond Chinese and English. Park and colleagues^47^ demonstrated that Arabic-English bilinguals tend to use a right-to-left chronological arrangement of cards illustrating different scenes from a story when using their L1 but not L2. Athanasopoulos and Bylund^57^ showed that Spanish-Swedish bilinguals tend to estimate time durations either as a quantity or as a distance, depending on whether they process temporal metaphors in their L1 (*small* vs. *big*) or L2 (*short* vs. *long*), respectively.

The studies reviewed above leave open the question of whether horizontal spatial biases accompany L2 temporal word processing. Moreover, only very few of these studies have controlled for L2 proficiency in their analyses—even though this is a key factor modulating the efficiency of L2 use and, therefore, also the potential activation of the MTL in L2. Existing research shows that higher L2 proficiency is associated with facilitated L2 lexical access^58^. Indeed, Athanasopoulos and Bylund^57^ demonstrated that the estimation of the duration of spatial configurations similar to those used in L2 time metaphors was modulated by L2 proficiency. Higher L2 proficiency was also shown to be associated with a stronger reliance on the vertical visuospatial mapping in L2 when it is different from the one common in L1^30^. At the same time, Yang et al.^59^ did not reveal any influence of L2 proficiency on space-time association in ME bilinguals, since all bilingual participants demonstrated MTL biases similar to Mandarin monolinguals. Therefore, while higher L2 proficiency may entail a stronger link between L2 words and respective spatial representations, existing evidence regarding the role of proficiency in the emergence of spatial biases in processing L2 words is mixed.

Importantly, studies using other conceptual domains, including *words related to power*, *spatial words*, *emotionally valenced words,* and *action-related words,* also show inconsistent results regarding sensorimotor activation in L2. On the one hand, several existing studies showed extremely rapid accrual of sensorimotor representational traces in novel word learning with even a brief exposure inducing stable sensorimotor effects for action words^60^ and for pseudowords denoting emotional valency^61^. Moreover, research showed that motor actions and physical interactions with objects facilitate rapid learning of novel concrete and abstract concepts^60,62–64^. This sensorimotor activation was shown to accompany adult L2 word processing, similar to L1 e.g.,^65,66^. At the neurophysiological level, activation in sensory and motor cortical areas was shown to accompany the processing of L1 and L2 words denoting graspable objects^53,67,68^. However, these studies differ vastly regarding *the balance* between the degree of reliance on sensorimotor processes in L1 and L2—while some studies report a similarly strong involvement of the attention and sensorimotor systems in L1 and L2^69^, others indicate differences between L1 than in L2^70,71^ which might be modulated by L2 proficiency^72^. Thus, even though the above findings suggest that attentional and sensorimotor traces accumulate already at the onset of language learning, their nature and persistence during the acquisition and use of L2 lexicon as well as their modulation by proficiency remain unclear. In the current study, we addressed these issues by (1) including participants with substantially different levels of L2 proficiency and (2) controlling for L2 proficiency in the statistical analysis.

Overall, the main goal of the present study was to investigate whether similar horizontal biases accompany access to time-related words in L1 and L2. Additionally, we aimed to evaluate the extent to which this putative signature of attentional engagement might be modulated by L2 proficiency. Below, we report the results of two experiments conducted on L2 English speakers with two different L1s: Russian in Experiment 1 and German in Experiment 2. All three languages have left-to-right oriented reading and writing direction, with English and German known to show a rightward MTL e.g.,^28^—English,^73^ – German. At the same time, there is only very limited evidence of horizontal space-time associations in Russian e.g.,^74^. Thus, the present research aimed to further extend existing evidence and generalize the corresponding findings to this previously understudied language. Importantly, we used different L1s and the same L2 in our experiments, thus allowing to generalize our findings from two L1s with rather different lexical and morphosyntactic organization. We also controlled for L2 proficiency in both experiments, which was evaluated objectively using the Cambridge General English Test as well as an L2-L1 translation task (see Procedure subsection below). In doing so, we went beyond the conventional studies that assess L2 proficiency merely by subjective self-reports e.g.,^30,55^.

In both experiments, we implemented a semantic time classification task—a method that has been widely used in MTL research^32,44,45^,e.g.,^75^. We chose this task because time-relevant tasks tend to show larger effect sizes than time-irrelevant ones^35^. In its classical version, participants categorize time-related words as related to the past or the future by pressing lateralized response keys. In line with previous findings, we expected to find the STEARC effect, revealed by RT facilitation in congruent conditions (combinations of past-related word + left response button/future-related word + right response button) compared to incongruent conditions (past-related word + right response button/future-related word + left response button). Specifically, we expected to register similar horizontal attention shifts in both Russian and German L1s and in English L2, since the horizontal spatial mapping is presumed to be the same for all three tested languages with the same left-to-right reading direction. We also entertained the following contrasting hypotheses:

1. The STEARC effect should be stronger in L1 than in L2, and it should be modulated by L2 proficiency since some studies showed differences in the strength of spatial biases in L1 vs. L2 e.g.,^72^ as well as a positive correlation between attentional and sensorimotor traces in L2 words and proficiency^30,57^,e.g.,^69^.
2. At the same time, some studies failed to find the influence of L2 proficiency on L2 word access^59^,e.g.,^71^. Moreover, several studies showed a rapid accrual of sensorimotor traces in novel word learning^60^,e.g.,^61^. Therefore, an alternative hypothesis suggests that the strength of the predicted STEARC effect should be similar in L1 and L2, without any modulation by L2 proficiency.

Considering power estimations reported in a study by Beracci and Fabbri^32^ investigating space-time association in a similar semantic time classification task, we relied on the average number of participants used in previous studies being ~ 41. Based on this, we recruited 53 participants for Experiment 1 and 48 participants for Experiment 2. All participants gave their informed consent for participation. The selection of the target population pipeline in both experiments was determined by the fact that the typical young population both in Germany and in Russia are the most active users of English as L2 across communication contexts—both in their professional and personal life. At the same time, further studies are necessary in order to examine the degree and the nature of spatial-conceptual mappings of time words in other age groups as well as in the cultures with different reading and writing scripts^14,28^.

### Ethical approval

All experimental protocols were approved by the HSE University research ethics committee which ensures that participants are treated according to the Declaration of Helsinki.

## Experiment 1

### Experimental design and materials

To investigate visualspatial grounding of temporal concepts in both L1 and L2, we presented Russian-English bilinguals with Russian (L1) and English (L2) temporal words. For each language, 12 past- (e.g., *yesterday, вчepa*) and 12 future-related words (e.g., *tomorrow, зaвтpa*) were selected, resulting in a full set of 24 stimuli (see Table 1). Note that L1 and L2 lists were equivalents but not full exact translations since there were some difficulties in translation (e.g., English words *forthcoming* and *impending* have the same Russian translation *пpeдcтoящий*). There were no reliable differences between past- and future-related words in terms of their length and frequency (see results of independent sample *t* tests in Table 2). Words were nouns, adverbs, adjectives, and participles. The words’ lexical class was included in the main analysis as a random effect (see Analysis subsection below).

**Table 1:** L1 and L2 stimuli in Experiments 1 and 2 with their respective temporal association ratings obtained in norming studies.

Language											
English L2 (Experiments 1 and 2)				Russian L1 (Experiment 1)				German L1 (Experiment 2)			
Time											
Past	*M*	Future	*M*	Past	*M*	Future	*M*	Past	*M*	Future	*M*
Bygone	1.6	Ahead	4.2	былoe	1.1	бyдyщee	4.9	ehemals	1.0	absehbar	4.0
Earlier	1.8	Foreseeable	4.6	вчepa	1.0	бyдyщий	4.8	einst	1.0	anstehend	4.7
Finished	1.4	Forthcoming	4.8	вчepaшний	1.3	вcкope	4.3	einstmals	1.0	bevorstehend	4.8
Formerly	2.0	Future	4.8	дaвнo	1.2	гpядyщee	4.6	gestern	1.0	demnächst	4.8
Lately	2.0	Imminent	4.8	издaвнa	1.3	гpядyщий	4.8	jüngst	1.2	kommend	4.8
Past	1.0	Impending	4.8	издpeвлe	1.2	зaвтpa	4.9	kürzlich	1.0	künftig	5.0
Preceding	2.0	Prospective	4.2	минyвший	1.1	зaвтpaшний	4.9	letztens	1.2	morgen	4.7
Previous	1.4	Shortly	4.6	нeдaвнo	1.3	нacтyпaющий	4.4	neulich	1.2	nachher	4.5
Previously	1.2	Soon	4.8	пoзaвчepa	1.0	плaниpyeмый	4.5	seinerzeit	1.3	nächstens	4.8
Recent	2.0	Subsequently	4.2	пpoшлoгoдний	1.0	пocлeзaвтpa	5.0	unlängst	1.7	übermorgen	5.0
Recently	1.6	Tomorrow	4.8	пpoшлoe	1.1	пpeдcтoящий	4.7	vergangen	1.2	zukunft	5.0
Yesterday	1.0	Upcoming	4.8	пpoшлый	1.1	cкopo	4.5	vorgestern	1.0	zukünftig	5.0
*Average*	1.5	*Average*	4.6	*Average*	1.1	*Average*	4.7	*Average*	1.1	*Average*	4.8

Stimuli included in Experiment 1 (English and Russian sets of words) and Experiment 2 (English and German sets of words). *M* values represent means of associatedness rating for each specific item over the subject group. *Averages* represent averaged associatedness rating over all stimuli in each list. Values were obtained from norming studies (5-point Likert scale) and represent a range from 1 (definitely past-related word) to 5 (definitely future-related word) rated by native speakers of each language.

**Table 2:** Characteristics (M and SDs) of the words included in the stimulus materials.

Language	Past	Future	Difference
English			
Lemma Frequency (lg10)	1.54 (0.75)	1.33 (0.67)	*t*(22) = 0.71, *p* = .487
Length in letters	7.42 (1.68)	8.33 (2.57)	*t*(22) = -1.03, *p* = .312

Russian			
Lemma Frequency (lg10)	1.47 (0.71)	1.58 (0.58)	*t*(22) = -0.44, *p* = .663
Length in letters	7.72 (2.02)	8.42 (2.27)	*t*(22) = -1.14, *p* = .267

German			
Lemma Frequency (lg10)	1.13 (0.51)	1.25 (0.55)	*t*(22) = -0.57, *p* = .577
Length in letters	7.83 (1.53)	8.33 (1.67)	*t*(22) = -0.76, *p* = .452

Results of independent-samples *t*-tests for past- vs. future-related groups of words used in Experiment 1 (English and Russian) and Experiment 2 (English and German).

### Preliminary norming study

In order to verify the intended temporal semantics of these words, we conducted separate norming studies for each list of words. Participants (separate from the main experimental group) indicated whether the word belongs to the future, past, or somewhere in between using a non-numerical computerized questionnaire with five response options ranging from "definitely past" to "definitely future". For statistical analysis, the responses were offline assigned scores on a 5-point Likert scale from 1 (definitely past) to 5 (definitely future). For the Russian list of words, we tested a sample of Russian native speakers (*N* = 20). The results indicated the following group averages: *M* = 1.1 for past-related words and *M* = 4.7 for future-related words (see Table 1 for details). For the English list of words, participants with English as L1 were recruited (*N* = 5). The results indicated *M* = 1.5 (past-related words) and *M* = 4.6 (future-related words) as group averages (see Table 1 for details). Thus, we concluded that these words indeed activate past and future projecting semantics as intended.

### Main experiment

All words were randomly presented in capital letters in the center of the screen. Participants were asked to classify them as related to the past or future by pressing the left or right response keys under instructions that rendered stimulus/response combinations as congruent and incongruent. Thus, the combinations of time-related words and response keys *past* + *left key* and *future* + *right key* were established as congruent experimental conditions, and *past* + *right key / future* + *left key*—as incongruent conditions. The dependent variable was Reaction Time (RT), defined as the time from word onset to button press in milliseconds. In addition, the participants were asked to perform the Cambridge General English Test, as well as a custom-made translation task (see below), to access their L2 Proficiency. As a result, the following experimental factors were independently manipulated in a within-subjects design: Language (L1/L2) and Congruency (Congruent/Incongruent). L2 Proficiency was included as a continuous predictor representing a score obtained from the Cambridge General English Test.

### Procedure

The experiment was implemented and hosted in Gorilla Experiment Builder^76^. Data were collected in 2021. In order to maximize the quality of the data, participants were asked to sit in a quiet and dimly lit room, place both the screen and the keyboard in the center with respect to themselves, close all irrelevant software tabs and windows, and switch to a full-screen mode. Before the main experiment, participants were asked to perform a short questionnaire with demographic items.

**Figure 1:**
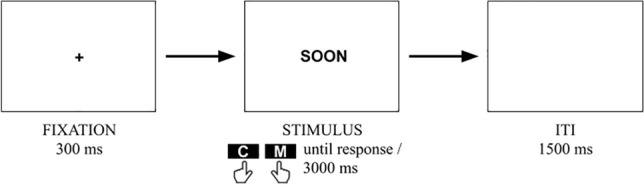
Example L2 stimulus and experimental trial sequence.

The experiment consisted of two blocks (randomized and counterbalanced) with a short break between them to reduce fatigue. At the beginning of their individual experimental sessions, participants were asked to rest their index fingers on the *c* and *m* buttons (US English layout) on a keyboard. Next, participants were instructed to read words in L1 or L2 and classify them as related to the past/future as fast and accurately as possible by pressing the lateralized response keys. The instruction was varied in each block: in the congruent block, left/right response keys corresponded to the past-/future-related words, respectively. In the incongruent block, the correspondence was reversed. Each block started with eight practice trials, consisting of stimuli that were not used in the main experiment. Each individual trial started with a centrally presented fixation cross that remained visible for 300 ms. After the fixation cross, a word appeared centrally on the screen (a past- or future-related word in L1 or in L2). The stimulus remained on the screen until a response key was pressed but no longer than 3000 ms. While previous research has demonstrated that even a short word presentation (e.g., 250 ms) is sufficient to activate time–space associations^73^, our task entailed additional cognitive demands, including stimulus-based decision-making. Consequently, we opted for a prolonged stimulus presentation duration, aligning with methodologies employed in similar studies^45,77^. Thus, we ensured that participants had sufficient time to fully process the stimuli, thereby enhancing the accuracy and reliability of their responses. Each trial ended with a 1500 ms inter-trial interval. Each element of the stimulus set was presented randomly, once in each of the two blocks. A typical trial sequence is shown in Fig. 1. If participants classified the word incorrectly or missed the response during the 3000 ms period, the trial was coded as incorrect.

After the time classification task, participants’ L2 proficiency was assessed with the help of the Cambridge General English Test. Finally, participants were presented with the full set of English words (24 stimuli) used in the experiment and asked to translate them into L1. If participants did not know the translation of the word, they could mark it in the appropriate box. If all words were translated correctly, participants received a Word Recognition Score equal to 24. This procedure was implemented in order to check whether participants understood all words while performing the task. In addition, the Word Recognition Score was used as an additional stimulus-specific measure of L2 Proficiency (see Results for detail).

### Participants

Fifty-three participants were recruited (40 females, mean age 27.5 ± 6.8 years) who did not take part in the norming study. As part of the selection criteria, participants had to be Russian native speakers and English second language learners (English should not be their second native language). Forty-eight participants reported themselves as right-handed. All participants reported having no diagnosis of dyslexia or other language deficits. Participants were recruited through the Internet (social media) and were remunerated for participation.

## Data preprocessing and analysis

Statistical analysis was executed using R software version 4.1.3^78^. Task-related RT was defined as the time interval between the stimulus onset and the provided response. For data trimming, incorrect responses and trials without responses (including delays over the 3000-ms timeout) were excluded. To normalize RT data, a reciprocal transformation was applied on the values (1/RT). A Linear Mixed Effects Model (LMM) analysis was then run on this measure using the *lme4* package^79^. Language (L1/L2), Congruency (Congruent/Incongruent), and L2 Proficiency (continuous) were included in the model as fixed effects. Interactions between all factors were also included. Subjects and stimuli were indicated as random effects. A continuous predictor (L2 Proficiency) was mean-centered. Categorical predictors (Language and Congruency) were assigned sum-coded contrasts (− 0.5 and 0.5)^80^. Backward elimination using the *drop1* function was employed to identify the best-fit model: effects and interactions that did not improve model fit (*p* > 0.1) were successively eliminated. For better readability of the model, 1 was subtracted from 1/RT values (in order to reverse signs in the output) and the obtained measures were multiplied by 1000 (in order to reduce the number of decimal places) (X = (1/RT − 1)*1000).

## Results

**Figure 2:**
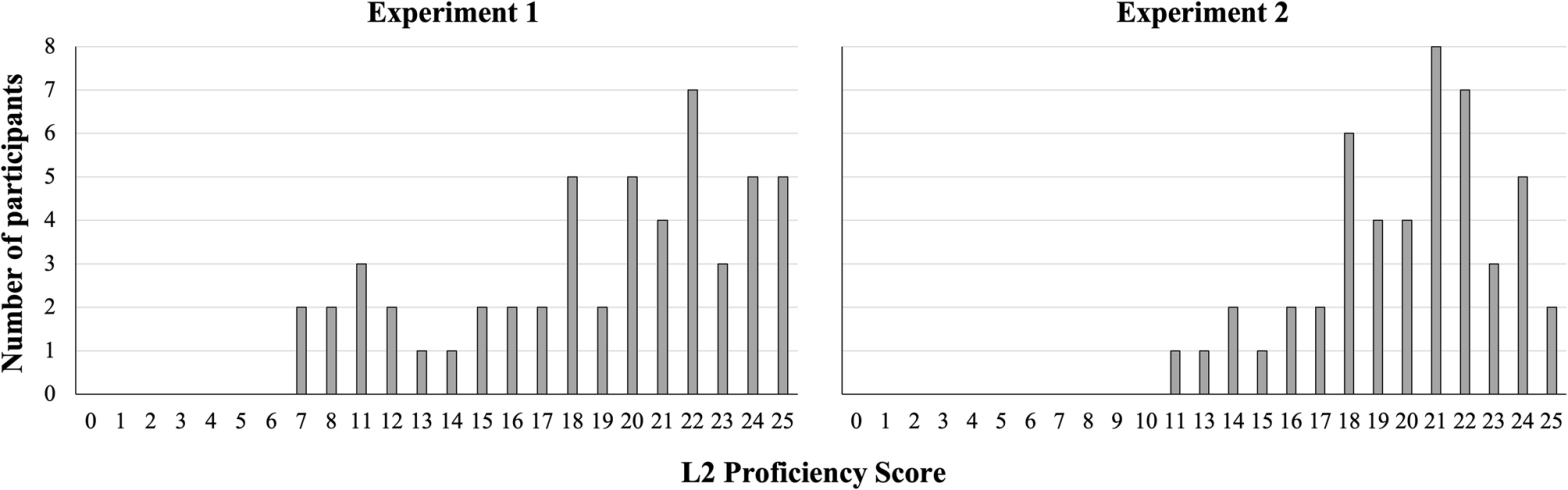
Distributions of L2 proficiency across the participant samples in Experiments 1 and Experiment 2. *Note:* X-axes represent scores obtained from Cambridge General English Test.

Participants’ L2 proficiency varied from 7 to 25 (*M* = 19, *SD* = 5.1; see Fig. 2, Experiment 1). After backward elimination, three effects (Language, Congruency, and L2 Proficiency) as well as an interaction between L2 Proficiency and Language remained in the model. Marginal *r*-squared (variance explained by fixed effects only, i.e., without random effects) was 0.137, and conditional *r*-squared (variance explained by the whole model) was 0.468. The statistical results (the output from the best-fit linear mixed-effects model) are presented in Table 3.

**Table 3:** Experiment 1. Model output from the best-fit linear mixed-effects model.

Random effects:	Name	*Variance*	*SD*	
Participants	Intercept	0.0244	0.1563	
Stimuli	Intercept	0.0261	0.1615	
Residual		0.0811	0.2847	

Fixed effects:	*b*	*SE*	*t*-value	*p*-value
Intercept	− 1.1789	0.0320	− 36.83	< .001
Language	0.2473	0.0474	5.22	< .001
L2 Proficiency	− 0.0141	0.0043	− 3.27	.001
Congruency	− 0.0766	0.0084	− 9.10	< .001
L2 Proficiency * Language	− 0.0073	0.0017	− 4.20	< .001

The following transformation of the dependent variable (RT, in ms) was performed: X = (1/RT − 1)*1000 (see Analysis subsection for details). As a result, *Variance*, *SD*, *b*, and *SE* columns in this output table are transformed. See the main text for raw values across conditions.

The analysis registered a reliable main effect of Language (b = 0.2473, p < 0.001): response RTs were faster for L1 (*M* = 845 ms, *SD* = 322) than for L2 (*M* = 1066 ms, *SD* = 469). The main effect of L2 Proficiency was also reliable (*b* = -0.0141, *p* = 0.001): the higher participants' L2 proficiency was, the faster they performed the task. Furthermore, the analysis revealed a reliable interaction between L2 Proficiency and Language (*b* = − 0.0073, *p* < 0.001), indicating smaller L1–L2 difference for more proficient participants. More importantly, the model yielded a significant main effect of Congruency (*b* = -0.0766, *p* < 0.001), suggesting faster response RTs in congruent conditions (*M* = 912 ms, *SD* = 392) in comparison with incongruent ones (*M* = 984 ms, *SD* = 428).

To further scrutinize this finding, we ran an additional LMM analysis with the same steps, procedure, and included factors, with one important distinction of decomposing the single main effect of Congruency into two separate effects of Time (past/future) and Response Key (left/right). This additional analysis fully confirmed the initial result above. Namely, it registered that participants responded faster to past-related words using the left key (*M* = 909 ms, *SD* = 356) than the right key (*M* = 980 ms, *SD* = 418) (*b* = -0.0879, *SE* = 0.0120, *t* = -7.32, *p* < 0.001). Similarly, they responded faster to future-related words using the right key (*M* = 916 ms, *SD* = 427) than the left key (*M* = 989 ms, *SD* = 440) (*b* = 0.0646, *SE* = 0.0118, *t* = 5.50, *p* < 0.001).

As an additional analysis, a Pearson’s correlation was computed on L2 Proficiency measure and Word Recognition Score in order to check the reliability of participants’ L2 proficiency obtained from the Cambridge General English Test in relation to the specific stimulus set. Participants’ Word Recognition Scores varied from 7 to 24 (*M* = 19, *SD* = 3.8). The analysis registered a reliable positive correlation (*r*(51) = 0.79, *p* < 0.001) between the two factors. Thus, the Cambridge General English Test reliably reflected participants’ L2 proficiency.

To summarize, Experiment 1 demonstrated a reliable main effect of Congruency between the key mapping and the mental time line representation, regardless of the language participants used: words denoting the past in either L1 or L2 were classified faster with the left key and words denoting the future in either L1 or L2 were classified faster with the right key. Moreover, L2 proficiency did not influence the strength of spatial biases in L2: There was no reliable interaction between L2 Proficiency and Congruency. Given that this is the first MTL research comparing L1 and L2, these novel results should be replicated and extended. Experiment 2 was conducted with these objectives.

## Experiment 2

### Experimental design and material

In Experiment 2, we presented German-English bilinguals with German (L1) and English (L2) time-related words. As in Experiment 1, 12 past- (e.g., *yesterday, gestern*) and 12 future-related words (e.g., *tomorrow, morgen*) were selected for each language. For the English list of words, the same stimuli were used as in Experiment 1. Again, it was impossible to create a list of exact equivalents of English words in German (e.g., *übermorgen* is a single lexeme in German, while in English it is three words: *the* *day after tomorrow*). For this reason, we created a separate list of German words that partially overlaps with the English list but also includes stimuli that do not appear in it. The full set of stimuli is presented in Table 1. There were no reliable differences between past- and future-related words in terms of letter length and frequency (see results of independent sample *t*-tests in Table 2). Words were nouns, adverbs, adjectives, and participles. Similar to Experiment 1, lexical class was included in the main analysis as a random effect (see Analysis for detail).

### Preliminary norming study

Following the same procedures as in Experiment 1, we conducted a separate norming study for the German list of words. German native speakers (*N* = 6) we asked to estimate the words as related to the past or the future using the same rating procedures as described for Experiment 1 above. The results indicated the following group averages: *M* = 1.1 for past-related words and *M* = 4.8 for future-related words (see Table 1 for details), highly similar to both the English L2 and Russian L1 items normed in Experiment 1. Thus, these words indeed activate past and future projecting semantics.

### Main experiment

As in Experiment 1, the stimulus set was presented randomly, once in each of the two blocks. All words were presented in capital letters. Participants were asked to perform a time classification task by pressing the left and right response keys. Participants’ objective L2 proficiency measure was also obtained by the Cambridge General English Test and custom-made translation task. The same experimental factors were independently manipulated in a within-subjects design: language (L1/L2), Congruency (Congruent/Incongruent), and L2 Proficiency was used as a continuous predictor.

### Procedure

The procedure was the same as in Experiment 1, except that the words were presented in German L1 and English L2. Data were collected in 2021.

### Participants

48 participants were recruited (33 females, mean age 24 ± 8.5 years). As part of the selection criteria, participants had to be German native speakers and English second-language learners (English should not be their second native language). 43 participants reported themselves as right-handed. All participants reported having no diagnosis of dyslexia or other language deficits. All participants were students at the University of Potsdam. Participants were recruited through the local Sona System (https://www.sona-systems.com) and received course credit for their contribution.

## Analysis

The analysis was identical to that employed in Experiment 1.

## Results

Participants’ L2 proficiency varied from 11 to 25 (*M* = 20, *SD* = 3.2; see Fig. 2, Experiment 2). After backward elimination, all effects as well as an interaction between L2 Proficiency and Congruency remained in the model. The statistical results (the output from the best-fit linear mixed-effects model) are presented in Table 4. Marginal *r*-squared was 0.102, and conditional *r*-squared was 0.470. Consistent with the pattern observed in Experiment 1, the analysis yielded a significant main effect of Language (*b* = 0.1026, *p* = 0.034): response RTs were faster for L1 (*M* = 918 ms, *SD* = 379) than for L2 (*M* = 990 ms, *SD* = 419). L2 Proficiency effect was also reliable (*b* = − 0.0348, *p* < 0.001): the higher the participants' L2 proficiency, the faster they performed the task. Again, the main effect of Congruency was reliable (*b* = − 0.0437, *p* < 0.001), reflecting faster RTs in congruent conditions (*M* = 933 ms, *SD* = 392) in comparison with incongruent ones (*M* = 970 ms, *SD* = 406). Finally, the model yielded a reliable interaction between L2 Proficiency and Congruency (*b* = − 0.0055, *p* = 0.049), suggesting that higher L2 Proficiency produced stronger space-time associations.

**Table 4:** Experiment 2. Model output from the best-fit linear mixed-effects model.

Random effects:	Name	*Variance*	*SD*	
Participants	Intercept	0.0280	0.1673	
Stimuli	Intercept	0.0271	0.1647	
Residual		0.0795	0.2819	

Fixed effects:	*b*	*SE*	*t*-value	*p*-value
Intercept	− 1.1729	0.0342	− 34.28	< .001
Language	0.1026	0.0484	2.12	.034
L2 Proficiency	− 0.0348	0.0076	− 4.56	< .001
Congruency	− 0.0437	0.0089	− 4.88	< .001
L2 Proficiency * Congruency	− 0.0055	0.0028	− 1.97	.049

The following transformation of the dependent variable (RT, in ms) was performed: X = (1/RT − 1)*1000 (see Analysis subsection for details). As a result, *Variance*, *SD*, *b*, and *SE* columns in this output table are transformed. See main text for raw values across conditions.

**Figure 3:**
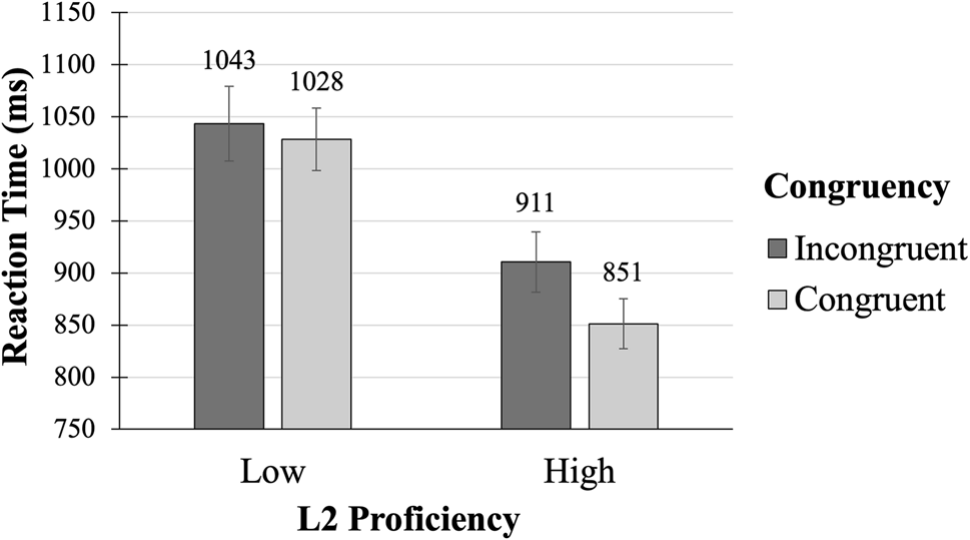
Experiment 2. Differences in congruency depending on L2 proficiency. *Note:* Interaction between L2 Proficiency (low/high) and Congruency (congruent / incongruent). Mean RTs in ms. Error bars represent standard errors.

To follow up on the interaction between L2 Proficiency and Congruency, further analysis was performed using linear mixed models. To this end, L2 Proficiency was first coded as a binary factor with two levels divided by median (*Median* = 21): low- (from 11 to 20; *N* = 23) and high- (from 21 to 25; *N* = 25) proficiency groups. As a new categorical predictor, it was assigned sum-coded contrasts (− 0.5 and 0.5). The data were then submitted to a linear mixed model analysis with the same factors and interactions between them as in the main analysis. When the data were split by L2 Proficiency group, the analyses revealed that participants with high L2 proficiency were significantly faster in congruent conditions (*M* = 851 ms, *SD* = 317) than in incongruent ones (*M* = 911 ms, *SD* = 366) (*b* = − 0.0698, *SE* = 0.0121, *t* = − 5.76, *p* < 0.001), with no such difference (*b* = − 0.0127, *SE* = 0.0132, *t* = − 0.96, *p* = 0.339) for participants with low L2 proficiency (congruent conditions: *M* = 1028 ms, *SD* = 444; incongruent conditions: *M* = 1043 ms, *SD* = 439) (see Fig. 3).

Scrutinizing the results pattern specifically for the high L2 proficiency group, we repeated the same additional LMM procedures and steps as described in Experiment 1, splitting the Congruency factor into two separate ones: time (past/future) and Response Key (left/right). This analysis registered faster reaction times when past-related words were combined with the left key (*M* = 871 ms, *SD* = 316) than with the right key (*M* = 929 ms, *SD* = 381) (*b* = − 0.0901, *SE* = 0.0173, *t* = -5.21, *p* < 0.001) and when future-related words were combined with the right key (*M* = 830 ms, *SD* = 319) than with the left key (*M* = 891 ms, *SD* = 349) (*b* = 0.0503, *SE* = 0.0170, *t* = 2.97, *p* < 0.005).

As in Experiment 1, an additional Pearson’s correlation analysis was performed on L2 Proficiency measure and Word Recognition Score to check the reliability of participants’ L2 proficiency obtained from the Cambridge General English Test. Participants’ Word Recognition Scores varied from 13 to 24 (*M* = 19, *SD* = 2.2). The analysis registered a reliable positive correlation (*r*(46) = 0.40, *p* = 0.005) between the factors. Thus, the Cambridge General English Test reliably reflected participants’ L2 proficiency with respect to the specific stimulus set.

Overall, the general effect of an association between temporal semantics and spatial responses (the STEARC effect) was observed in both L1 and L2, since there was no reliable interaction between Congruency and Language. This is consistent with the results obtained in Experiment 1, thereby replicating and extending this novel finding. At the same time, the significant interaction between Congruency and L2 Proficiency reflected that the general spatial congruency effect was only reliable in the high-proficiency group, but not in the low-proficiency group. Thus, the strength of the spatial biases did depend on participants’ L2 proficiency. This observation also provides a possible explanation for the inconsistent findings in previous studies, as we will discuss below.

## General discussion

Two experiments reported in this paper aimed to examine whether access to time-related words is accompanied by activation of the horizontal MTL in both L1 and L2. The additional goal was to investigate whether the strength of this activation is modulated by L2 proficiency. For this purpose, we asked Russian-English (Experiment 1) and German-English (Experiment 2) bilinguals to read temporal words in their L1 and L2 and classify them as related to the past or the future by pressing left and right response keys in a counterbalanced fashion. We investigated shifts in visual attention caused by processing these words, registered as RT differences in congruent (past + left, future + right) vs. incongruent (past + right, future + left) conditions. In addition, participants’ L2 proficiency was assessed by an internationally established linguistic tool, the Cambridge General English Test, as well as by a custom-made translation task. To the best of our knowledge, this study is the first that compares access to time-related words in both L1 and L2 with respect to the activation of the horizontal MTL. This represents a novel contribution to the field, expanding our understanding of bilingual cognitive processing in the context of temporal language. Furthermore, this study included participants with various levels of L2 proficiency, and the statistical analysis specifically controlled for this parameter.

Confirming our first hypothesis, we found the STEARC effect in both experiments, indicating the activation of the horizontal MTL during the processing of time-related words. These findings are consistent with previous research showing a horizontal shift in visual attention for past- and future-related words in L1^32,44,45,75^. More specifically, the obtained results are in line with the studies showing horizontal time–space association in German e.g.,^73,81^, English e.g.,^28^, and with limited reports of a horizontal MTL in Russian^74^. Thus, our findings further extend the generalizability of the resulting theoretical inferences by offering evidence from both German and Russian as native languages.

More importantly, we revealed the STEARC effect in L2. This pattern was consistent in both our experiments: Participants were faster in congruent conditions than in incongruent ones regardless of the language they used. We can conclude that these L2 STEARC phenomena are sufficiently robust since we successfully replicated them across late bilinguals in two experiments with different L1s. Finding similar left-to-right oriented MTL across Russian, German, and English might be explained by cultural effects, e.g., left-to-right reading and writing systems shared across all three tested languages. The emergence of the STEARC effect in these three languages confirms our hypothesis of a consistent horizontal spatial mapping of temporal concepts. Moreover, the results are in line with research supporting the notion of spatial biases accompanying access to temporal semantics in L2^26,30,55–57^. Our findings are also consistent with previous results showing reliable associations between space and other concepts in L2, e.g., an association between vertical space and spatial words e.g.,^69^, emotionally-valenced words e.g.,^82^, and words related to power^72^.

Furthermore, we obtained some evidence regarding our competing hypotheses about the modulation of the strength of the STEARC effect by L2 proficiency: Higher L2 proficiency was associated with stronger spatial biases in German-English bilinguals whereby the STEARC effect was registered only in the highly proficient group, without reliable differences between experimental conditions in the low proficient group. However, there was no such effect in Experiment 1: Russian-English bilinguals showed equally strong STEARC effect regardless of their level of L2 proficiency. This inconsistency could reflect differences in L2 proficiency across two samples; however, we recruited participants with practically equal ranges of L2 proficiency in both experiments, with an even narrower range in Experiment 2: from 7 to 25 for Russian-English bilinguals and from 11 to 25 for German-English bilinguals. Another explanation could be the difference in L2 proficiency distributions, with unequal distribution across high- and low-levels compromising statistical comparisons. However, the distribution of L2 proficiency in Experiment 2 was even less heterogeneous than in Experiment 1, with a larger percentage of highly proficient participants (see Fig. 3) which speaks against this explanation.

Notably, the results of previous studies with variable L2 proficiency have also yielded inconsistent results see for positive findings^30,69,72^, but see for null findings^59,70,71^. Therefore, one possible explanation might be the influence of other L2 parameters, e.g., age of L2 acquisition (AoA), context of L2 acquisition and use, etc.^52^. One can assume that early bilinguals (those who began acquiring L2 during their childhood, typically before 6 years of age) might have a stronger overlap in semantic information between L1 and L2. Thus, sensorimotor and attentional systems in early bilinguals should be involved in L2 semantic access to a degree more similar to that in L1. To support this, one previous study^26^ showed a positive correlation between L2 AoA and the tendency to use vertical space-time association in ME bilinguals but see^70^ for controversial results in action words. In our study, differences in the influence of L2 proficiency might be also explained by participants’ AoA (or potentially some other unaccounted-for factors), which should be further explored and verified in future investigations.

The contexts of acquisition and use also might modulate differences in the degree of grounding of language processing in sensorimotor and attentional systems. Generally, L1 is acquired in natural conditions requiring ongoing wide-range interactions with the external world via the body and activation of somatosensory systems. Unlike L1, L2 is typically acquired in schools and work environments through abstract formal instruction and without much direct sensorimotor experience. Although we could not control for these parameters, one might assume that German-English bilinguals use their L2 in a wider set of contexts (since it is one of the main EU languages used in tuition, travel, and daily interactions, given the massive expat population in Germany) than Russian-English bilinguals who use their L2 primarily in professional and academic environments, and almost never in everyday life which is dominated by L1. Finally, an influence of other L2 types, especially those with different reading and writing directions, could affect our findings as well e.g.,^48,49^. Therefore, future studies should both control for the diverse L2 features mentioned above and include them in statistical analyses. Furthermore, a more comprehensive approach to assessing L2 proficiency, e.g., estimating both language comprehension (e.g., DIALANG) and production (e.g., Productive Vocabulary Levels Test) might also be beneficial.

Considering the findings and observations presented in this study, another intriguing direction for future research emerges. While we effectively controlled for the unique characteristics of each stimulus by LMM analyses, future studies might investigate potential cross-linguistic differences in reaction times between words that are more abstract and distant to the present (e.g., “recent”, “soon”, “lately”) and those that denote temporal concepts more concrete and closer to the present (e.g., “yesterday”, “tomorrow”) similar to^83,84^. Also, future research might document the timecourse of sensorimotor activation (e.g., by mouse tracking, eye tracking, or EEG/MEG) to investigate differences in the unfolding temporal dynamics of sensorimotor effects between L1 and L2. Finally, future research might consider potential variability in horizontal spatial-conceptual mapping across individuals, e.g., by employing both linguistic and nonlinguistic stimuli to establish baseline RTs for response differences in congruent vs. incongruent conditions (We thank the anonymous reviewer for this suggestion.).

To summarize, our findings provide general support for the notion of spatial biases accompanying access to time-related words in both L1 and L2. Moreover, we demonstrated the robustness of these results by successfully replicating them across two different L1 samples. To our knowledge, this is the first study addressing the question of whether horizontal spatial biases accompany L2 temporal word processing. We also found that special biases in L2 temporal words might positively correlate with L2 proficiency, although this connection was statistically reliable only in Experiment 2, which warrants further investigations. Overall, the present results support the notion of horizontal spatial biases of abstract time concepts in both native (L1) and second (L2) languages.

## Data Availability

We report in detail information regarding sample size, data exclusion, all experimental manipulations, and all the experimental measures across the two experiments. All the data and the analysis codes are publicly available via this link. Data in both experiments were analyzed using R package, version 4.1.3 (R Core Team, 2022). We did not preregister the experiments’ designs or analyses. The data for both experiments were collected in 2021.
